# HS-133, a novel fluorescent phosphatidylinositol 3-kinase inhibitor as a potential imaging and anticancer agent for targeted therapy

**DOI:** 10.18632/oncotarget.2507

**Published:** 2014-10-08

**Authors:** Ju-Hee Lee, Kyung Hee Jung, Hyunseung Lee, Mi Kwon Son, Sun-Mi Yun, Sung-Hoon Ahn, Kyeong-Ryoon Lee, Soyoung Lee, Donghee Kim, Sungwoo Hong, Soon-Sun Hong

**Affiliations:** ^1^ Department of Drug Development, College of Medicine, Inha University, Incheon, Republic of Korea; ^2^ Drug Discovery Platform Technology Team, Division of Drug Discovery Research, Korea Research Institute of Chemical Technology, Daejeon, Republic of Korea; ^3^ Center for Catalytic Hydrocarbon Functionalizations, Institute for Basic Science (IBS) and Department of Chemistry, Korea Advanced Institute of Science and Technology (KAIST), Daejeon, Republic of Korea

**Keywords:** Anticancer, Targeted therapy, PI3K, Fluorescence, Imaging, Apoptosis

## Abstract

As PI3K/Akt signaling is frequently deregulated in a wide variety of human tumors, PI3K inhibitors are an emerging class of drugs for cancer treatment. The monitoring of the drug behavior and distribution in the biological system can play an important role for targeted therapy and provide information regarding the response or resistance to available therapies. In this study, therefore, we have developed a family of xanthine derivatives, serving as a dual function exhibiting fluorescence, as well as inhibiting PI3K. Among them, HS-133 showed anti-proliferative effects and was monitored for its subcellular localization by a fluorescence microscopy. HS-133 suppressed the PI3K/Akt pathway and induced cell cycle arrest at the G0/G1 phase. The induction of apoptosis by HS-133 was confirmed by the increases of the cleaved PARP, caspase-3, and caspase-8. Furthermore, HS-133 decreased the protein expression of HIF-1α and VEGF, as well inhibited the tube formation and migration of the human umbilical vein endothelial cells. *In vivo* imaging also showed that tumors were visualized fluorescent with HS-133, and its oral administration significantly inhibited the growth of tumor in SkBr3 mouse xenograft models. Thus, we suggest that HS-133 may be used as a fluorescent anticancer agent against human breast cancer.

## INTRODUCTION

Phosphatidylinositol 3-kinases (PI3K) are responsible for the generation of 3-phosphorylated inositides, such as the important second messenger phosphatidylinositol-3, 4, 5-trisphosphate (PIP3), resulting in an activation of the signal transduction pathways implicated in many essential cellular processes, including metabolism, cell survival, growth, and differentiation [1–4]. The PI3K pathway is initiated by the binding of growth factors or insulin to the cell surface receptors, which activates the class IA PI3K that converts the plasma membrane lipid phosphatidylinositol-4, 5-bisphosphate (PIP2) to PIP3. PIP3 plays a key role in recruiting proteins, such as the serine/threonine kinases phosphoinositide-dependent kinase 1 (PDK1) and Akt (also known as PKB), to the plasma membrane [3, 5]. This in turn activates Akt which subsequently phosphorylates mTOR and several other cytoplasmic proteins [6]. A serine/threonine kinase, mTOR, has diverse cellular functions, including regulation of mRNA translation, cell growth and proliferation, ribosome biogenesis, transcription, cytoskeletal reorganization, long-term potentiation and autophagy [7]. The mTOR serves as the catalytic subunit in two distinct complexes, mTOR complex 1 (mTORC1) and mTOR complex 2 (mTORC2), which vary both in their subunit components and their function [8, 9].

The PI3K/Akt signaling pathway is the most commonly activated pathway in human cancer [6, 10, 11]. In particular, members of class IA PI3Ks among several isoforms of the PI3K family are implicated and often mutated in human cancer [12–15]. The deregulation of this pathway has been implicated in tumor initiation, cell growth and survival, invasion and angiogenesis [4]. As such, there is a strong rationale for targeting PI3K, especially in cancers that are known to carry alterations in PI3K/Akt signaling. Inhibition of this pathway is an attractive target for the development of new anticancer strategies. A new generation of PI3K inhibitors is emerging, overcoming earlier problems of poor selectivity, unfavorable pharmacokinetic profiles, and unacceptable toxicity [10, 16]. A number of these agents have entered the early phase clinical trials [17]. Some major pharmaceutical companies are currently testing the hypothesis that ATP competitive mTOR or dual-PI3K/mTOR inhibitors will be able to overcome the limited clinical responses that have been observed with rapamycin and its analogs (rapalogs) [18].

The monitoring of the drug behavior and distribution in the biological system can play an important role for the targeted therapy and provide a biological rationale for the design of new therapeutics with improved properties and fewer side effects [19]. A response to PI3K inhibition is often associated with tumor stasis rather than shrinkage [20–23]. The utility of such traditional imaging methods as computed tomography (CT), ultrasound imaging, and magnetic resonance imaging (MRI) in monitoring early response is limited [23]. Accordingly, there is a critical need for noninvasive functional imaging biomarkers that confirm drug delivery and molecular drug activity. The tethering of fluorescent dyes to drugs is a popular method for visually monitoring the time course of the drug behavior, and also provides a useful tool for studying cellular, animal and clinical imaging in a noninvasive, nonradioactive, and stable procedure [24–26]. However, the addition of fluorophores into the drug molecules often causes undesired effects on the binding affinity, cell permeability, *in vivo* activity and toxicity. These problems can be overcome if one drug has a dual function that exhibits fluorescence as well as anticancer activity.

Xanthines are known as important alkaloids, which are biologically active and constitute a major class of adenosine receptor antagonists, as well as fluorophores. We recently reported on the identification of a family of potent fluorescent PI3Kα inhibitors from xanthine scaffold in which the part of the fluorophore was engineered to be a pharmacophore capable of inhibiting PI3Kα [26, 27]. Further we showed that the xanthine derivatives blocked cancer cell proliferation and monitored its subcellular localization by fluorescent microscopy [26].

In the study, we selected *N*-(4-(7-(3-Fluorophenyl)-1, 3-dimethyl-2, 6-dioxo-2, 3, 6, 7-tetrahydro-1*H*-purin-8-yl)phenyl) acetamide (HS-133), which was found to be the most potent fluorescent PI3K inhibitor and investigated whether or not HS-133 possesses anti-cancer activity against breast cancer and its molecular mechanism. HS-133 potently inhibited PI3K/Akt pathway signaling and cell growth/proliferation in human breast cancer cells together with suppressing tumor growth in the xenograft models. Furthermore, the inherent fluorescence of HS-133 was detected in tumor bearing mice, as well as intracellular localization, and its pharmacokinetic properties exhibited good oral bioavailability.

## RESULTS

### Synthesis and binding mode of a new PI3Kα inhibitor, HS-133

**Figure 1:**
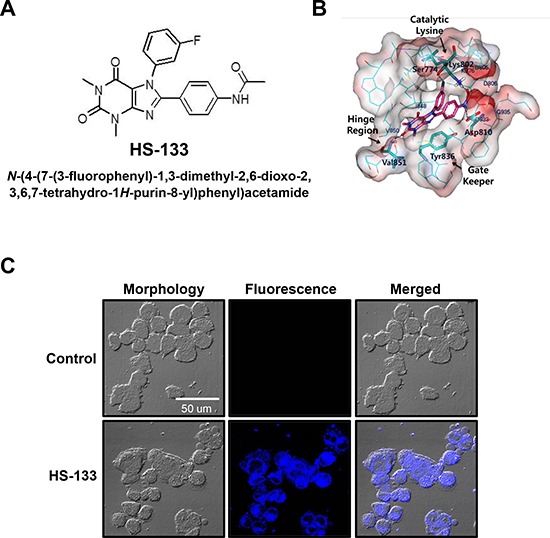
Chemical structure and characterization of HS-133 **(A)** Chemical structure of HS-133 [N-(4-(7-(3-fluorophenyl)-1,3-dimethyl-2,6-dioxo-2,3,6,7-tetrahydro-1H-purin-8-yl) phenyl) acetamide]. **(B)** Calculated binding mode of HS-133 in the ATP-binding site of PI3Kα. Carbon atoms of the protein and the ligand are indicated in cyan and magenta, respectively. Each dotted line indicates a hydrogen bond. **(C)** Confocal microscopic observation of the intracellular HS-133 (10 μM) disposition in SkBr3 cells.

A novel compound HS-133 was synthesized and was found to be a potent ATP-competitive inhibitor against PI3Kα (Fig. 1A) [26]. The calculated binding mode of the inhibitor HS-133 in the ATP-binding sites of PI3Kα was calculated using the modified Auto Dock program in Fig. 1B. Hydrogen bonding groups on the inhibitors appeared to point toward the backbone groups of the ATP binding site, with their hydrophobic groups situated in the proximity of the Gly loop. The carbonyl moiety at the 3-position of xanthine core can forms a hydrogen bond to the backbone aminocarbonyl nitrogen of Val851 in the hinge region, and the amide group of HS-133 donates a hydrogen bond to the side-chain carboxylate of Asp810. In addition, the fluoro group at the 5-phenyl group appeared to form a hydrogen bond with the hydroxyl group of Ser774. These three hydrogen bonds appear to play a role of anchoring HS-133 at the ATP-binding site. Compound HS-133 can be further stabilized in the ATP-binding site of the PI3Kα via hydrophobic interactions with the side chains of Lys776, Asp805, Asp806, Asp806, Tyr836, Ile848, Asp933, and Gly935. Thus, the overall structural features derived from the docking simulations suggested that the inhibitory activity of HS-133 should stem from multiple hydrogen bonds and hydrophobic interactions established simultaneously in the ATP-binding site.

### Intracellular monitoring studies by confocal microscopy

When SkBr3 breast cancer cells were incubated with HS-133 (10 μM) for 4 h, fluorescence became clearly visible in the cytoplasm of SkBr3 cells, providing visual evidence of the compound entering cells and information on the intracellular distribution pattern (Fig. 1C). Furthermore, at 24 h after the treatment, fluorescence images of the cells revealed severe morphological changes such as shrinkage, elongation, or a disorder in cell shape (data not shown), suggesting that this approach can also be useful for monitoring the antiproliferative processes in cancer cells associated with drug retention and concentration as a function of time.

### HS-133 blocks the PI3K/Akt pathway in breast cancer cells

**Figure 2:**
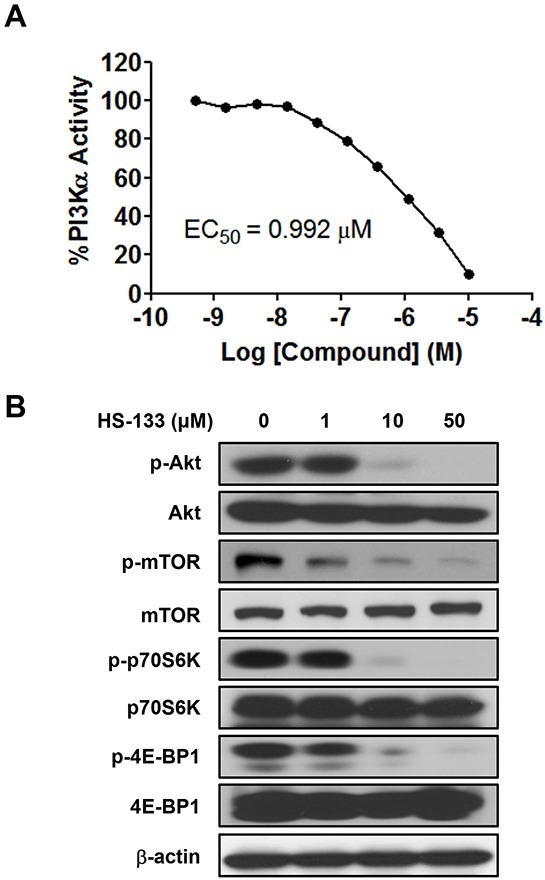
Effect of HS-133 on PI3K/Akt signaling pathway in SkBr3 cells **(A)** The inhibitory activity of HS-133 against PI3Kα *in vitro.* **(B)** Western blotting analysis in SkBr3 cells treated with HS-133 at various doses (1–50 μM) for 6 h.

It has been shown that breast tumor cell growth is closely related to the activation of the PI3K/Akt pathway [28–30]. The IC_50_ determination was performed using radiometric kinase assays ([γ-^33^P]-ATP) at the Reaction Biology Corp. (Malvern, PA), which revealed high potency against PI3Kα with an IC_50_ of 0.992 μM (Fig. 2A). Therefore, we evaluated the effects of HS-133 on the PI3K/Akt pathway in breast cancer cells. The PI3K inhibitory activity of HS-133 was assessed in the SkBr3 cells. The phosphorylation of Akt and its substrate, mTOR, and phosphorylation of downstream factors, including p70S6K and 4E-BP1were also effectively suppressed (Fig. 2B).

### HS-133 inhibits the growth of breast cancer cells

**Figure 3:**
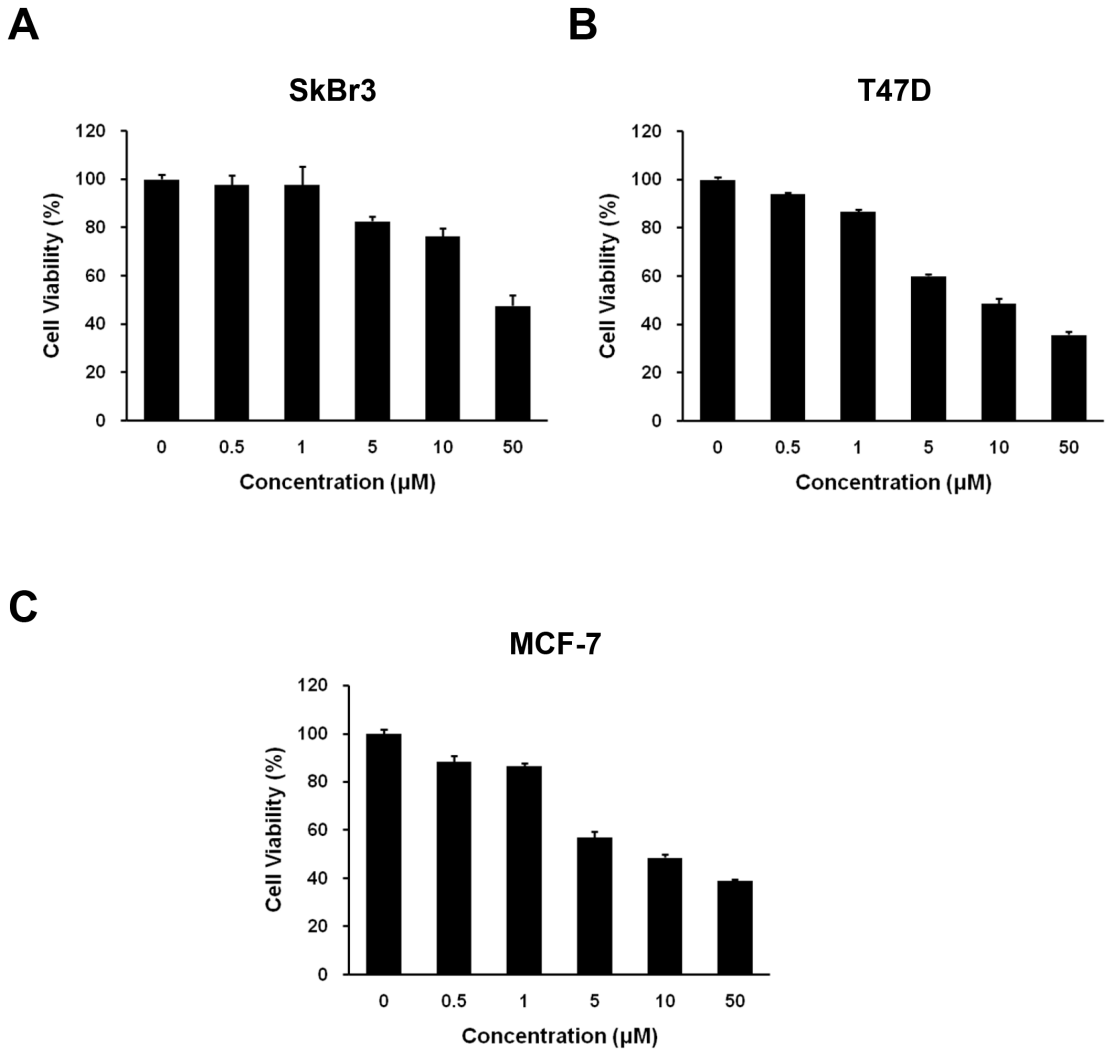
Effect of HS-133 on the proliferation of human breast cancer cells Inhibitory effect of HS-133 on cell proliferation in SkBr3 **(A)** T47D **(B)** and MCF-7 **(C)** Cell growth inhibition was assessed by MTT assay. Each value is the mean (±S.D.) from triplicate samples.

To determine whether HS-133 can function as a novel anticancer compound, we tested the ability of this compound to inhibit the growth of breast cancer cell lines (SkBr3, T47D, and MCF-7) using an MTT assay. The cells were exposed to various concentrations (0.5–50 μM) of HS-133 for 48 h. As shown in Fig. 3A-C, HS-133 inhibited the growth of all three breast cancer cell lines in a dose-dependent manner. The IC_50_ values of HS-133 were 46.76 μM for SkBr3, 9.4 μM for T47D, and 9.15 μM for MCF-7.

### HS-133 induces cell cycle arrest at G0/G1 phase and apoptosis

**Figure 4:**
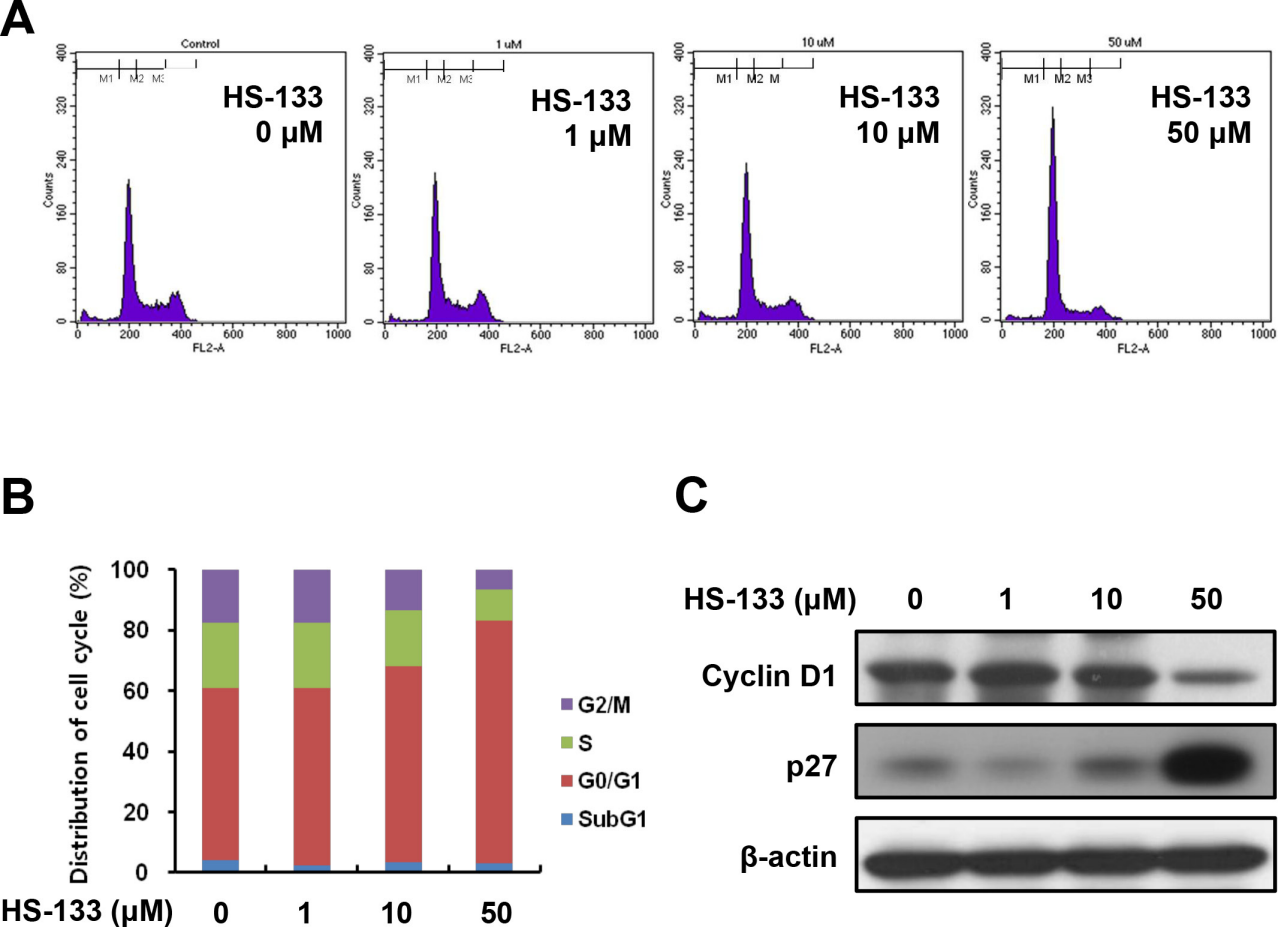
Effect of HS-133 on cell cycle in SkBr3 cells **(A)** SkBr3 cells were treated with HS-133 (1–50 μM) for 24 h, stained with propidium iodide (PI) and analyzed on a FACScalibur flow cytometer. **(B)** Quantification of the PI staining data was presented as the percentages of cell cycle distribution. **(C)** Western blotting of cell cycle-related proteins.

To examine the relationship between the growth inhibitory effect of HS-133 and cell cycle progression, we analyzed cell cycle distribution of SkBr3 cells by HS-133 using a flow cytometry. The SkBr3 cells were treated for 24 h with various concentrations of HS-133 (1, 10, 50 μM) and collected and stained with PI and then analyzed by FACS. As shown in the Fig. 4A and 4B, HS-133 increased the number of cells in the G0/G1 phase. The up-regulation of p27, cyclin dependent kinase (CDK) inhibitor, is related to G0/G1 cell cycle arrest and contributes to the down-regulation of cyclin D1 [31]. Thus, we evaluated the effect of HS-133 on the protein expressions of p27 and cyclin D1. HS-133 increased the expression of p27 while decreasing cyclin D1 expression (Fig. 4C).

**Figure 5:**
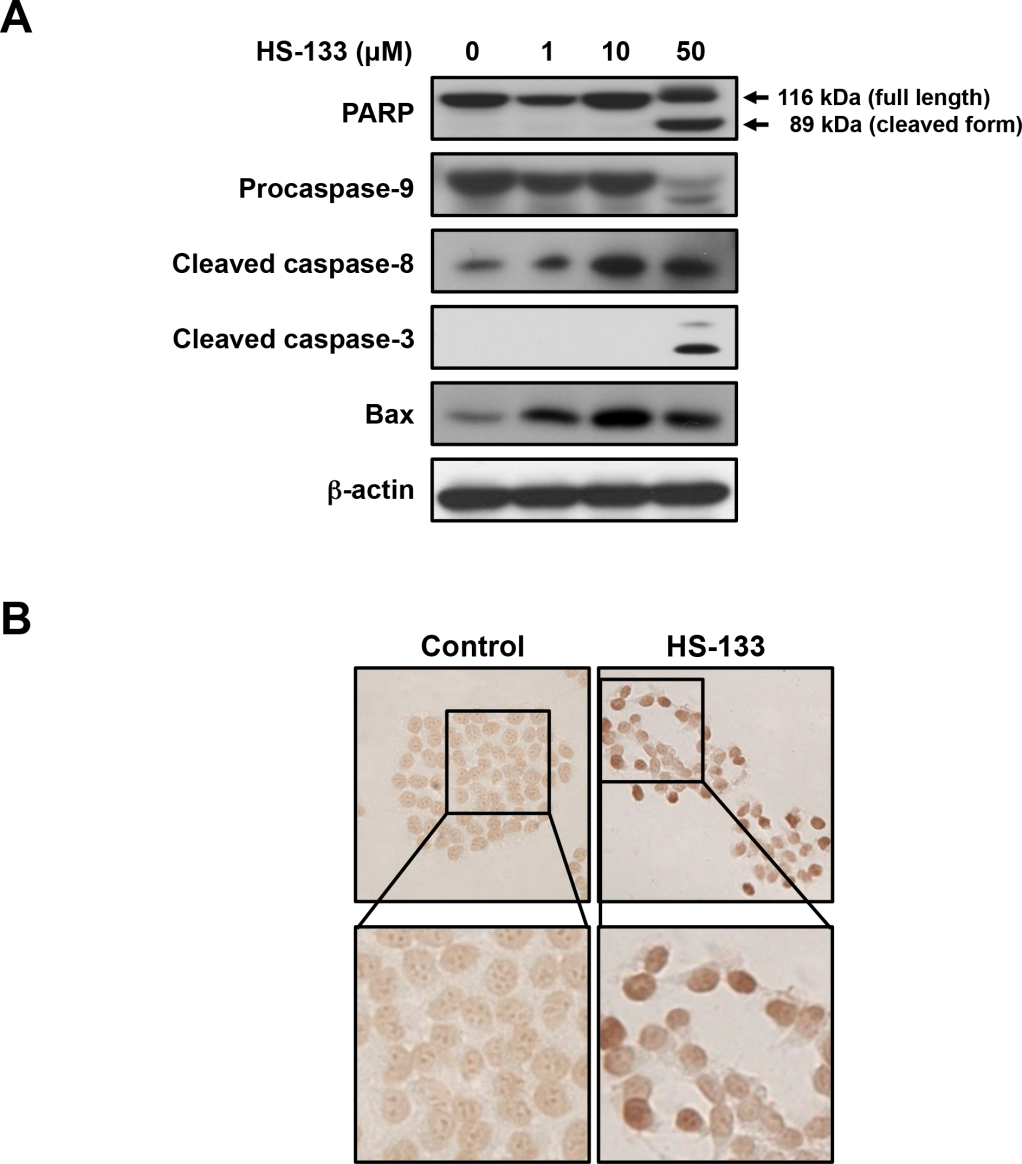
Effect of HS-133 on apoptosis in SkBr3 cells **(A)** The expression of the PARP, pro-caspase-9, cleaved caspase-8, cleaved caspase-3, and Bax were assayed by Western blotting in cells treated with HS-133 at the indicated doses for 72 h. **(B)** TUNEL assay in SkBr3 cells treated with or without HS-133 (50 μM). Cells treated with HS-133 at the indicated doses for 24 h.

Since PI3K and its downstream effectors appear to mediate anti-apoptotic signals, we examined the effect of HS-133 on apoptosis. As shown in Fig. 5A, HS-133 promoted the cleavage of PARP, caspase-9, caspase-8 and casepase-3. We also observed that the levels of Bax, pro-apoptotic protein, were increased by HS-133 treatment in a dose-dependent manner. Furthermore, induction of apoptosis by HS-133 was confirmed by visualizing the fragmented DNA with TUNEL staining (Fig. 5B). These results showed that HS-133 could induce apoptosis in the SkBr3 cells.

### HS-133 suppresses angiogenesis

**Figure 6:**
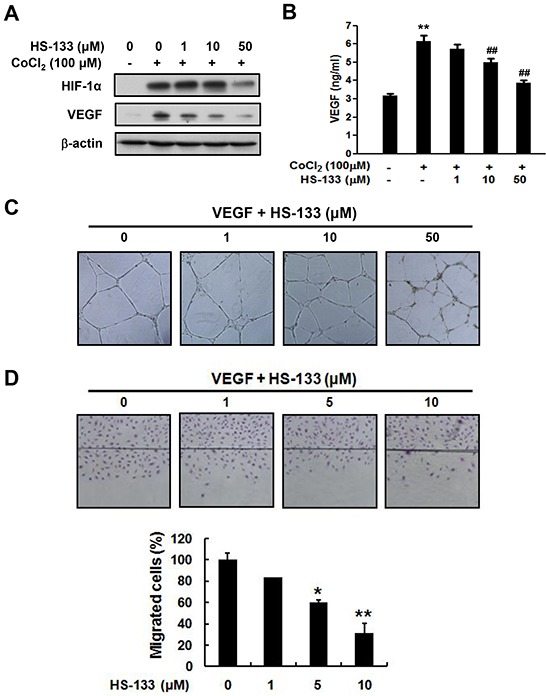
Effect of HS-133 on angiogenesis of SkBr3 cells and HUVECs **(A)** Expression of HIF-1α and VEGF by HS-133 in hypoxia-induced SkBr3 cells. The cells were treated with various concentrations of HS-133 (1–50 μM) under hypoxic mimicking conditions induced by a treatment with 100 μM CoCl_2_ for 16 h. **(B)** Production of VEGF by HS-133 in hypoxia-induced SkBr3 cells for 24 h. Statistical significance of differences between each treatment group and the control (**, p < 0.01) or CoCl_2_ alone (^##^, p < 0.01) was determined. Effects of HS-133 on tube formation **(C)** and migration assay (D) in HUVECs. To evaluate inhibitory effect of HS-133 on tube formation of HUVECs, the cells were treated with HS-133 of the indicated dose for 20 h. Wound migration assay was determined at indicated doses for 18 h. Statistical significance of differences between HS-133 treated groups and the control (*p < 0.05,**p < 0.01) was determined.

Since HIF-1α is the major regulator of cellular adaptive responses to hypoxia, we examined the effect of HS-133 on the expression of the HIF-1α in SkBr3 cells. The cells were treated with various concentrations of HS-133 (1–50 μM) under hypoxic mimicking conditions induced by a treatment with 100 μM CoCl_2_ for 16 h. As shown in Fig. 6A, HS-133 inhibited the hypoxia-induced HIF-1α expression. To further determine the effect of HS-133 on hypoxia-induced VEGF, an immediate downstream target gene of HIF-1α, the protein and production of VEGF were measured by western blotting and ELISA in the SkBr3 cells. A notable increase of VEGF was observed under hypoxic conditions, and the HS-133 treatment was found to suppress the VEGF expression and production in a dose-dependent manner (Fig. 6A and 6B).

To examine the effect of HS-133 on angiogenesis, a capillary tube formation assay using human umbilical vein endothelial cells (HUVECs) was performed. HS-133 inhibited the formation of vessel-like structures, characterized by elongation and alignment of the cells at the indicated concentrations (Fig. 6C). Cell migration is critical for the endothelial cells to form blood vessels during angiogenesis, and is necessary for tumor growth and metastasis. Thus, we conducted a wound migration assay to study the effect of HS-133 on cell migration. When the endothelial cell layer was wounded and incubated in a medium containing 1, 5, or 10 μM HS-133 for 18 h, the cell migration was significantly inhibited (Fig. 6D). Considering that endothelial migration and tube formation have all highly relevant properties of angiogenesis, our results illustrated that HS-133 has the ability to block angiogenesis.

### *In vivo* imaging of HS-133

**Figure 7:**
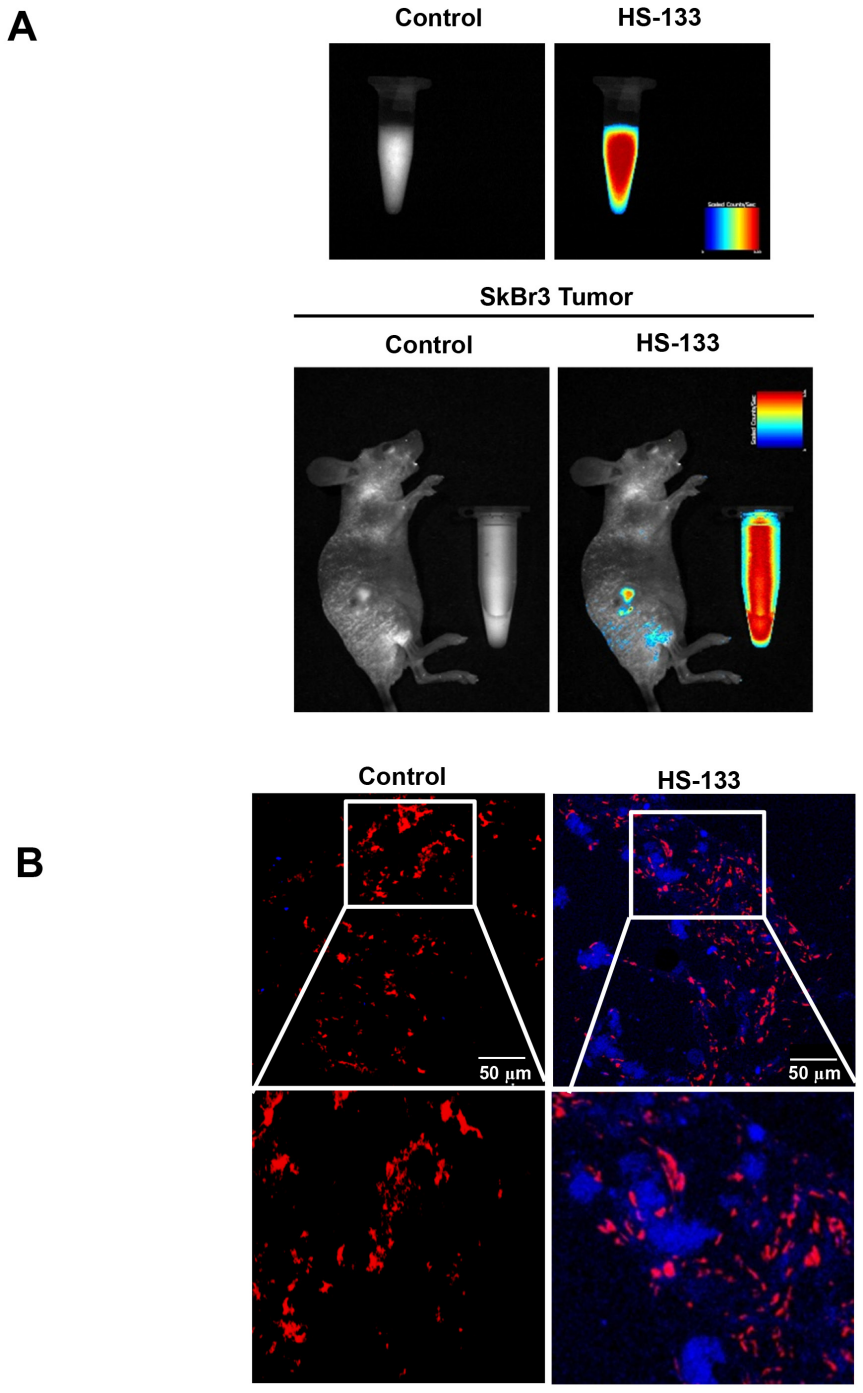
*In vivo* imaging of HS-133 **(A)** Images of a fluorescent HS-133 (50 mM) in solution and the intratumoral HS-133 disposition in SkBr3 xenograft models using the Maestro™ In-Vivo Fluorescence Imaging System. **(B)** Confocal observation of tumors isolated 1 h after the injection of vehicle or 5 mg/kg HS-133 (Blue) into SkBr3 xenograft models and observed with a confocal laser scanning microscope after propidium iodide (PI, Red) staining.

To evaluate whether HS-133 can be detected as fluorescent in the tumor, we used the SkBr3 xenograft model in which human breast cancer cells were inoculated into the dorsal flank of BALB/c nude mice. Fluorescence of HS-133 was obviously detected when HS-133 was injected intratumorally into SkBr3 tumor-bearing mice (Fig. 7A). Tumors were excised at 1 h after the intratumoral injection of HS-133, frozen sectioned, and observed with a confocal laser scanning microscope after propidium iodide (**PI)** staining. As a result, the fluorescence by HS-133 became clearly visible in the isolated tumor (Fig. 7B).

### HS-133 inhibits tumor growth

**Figure 8:**
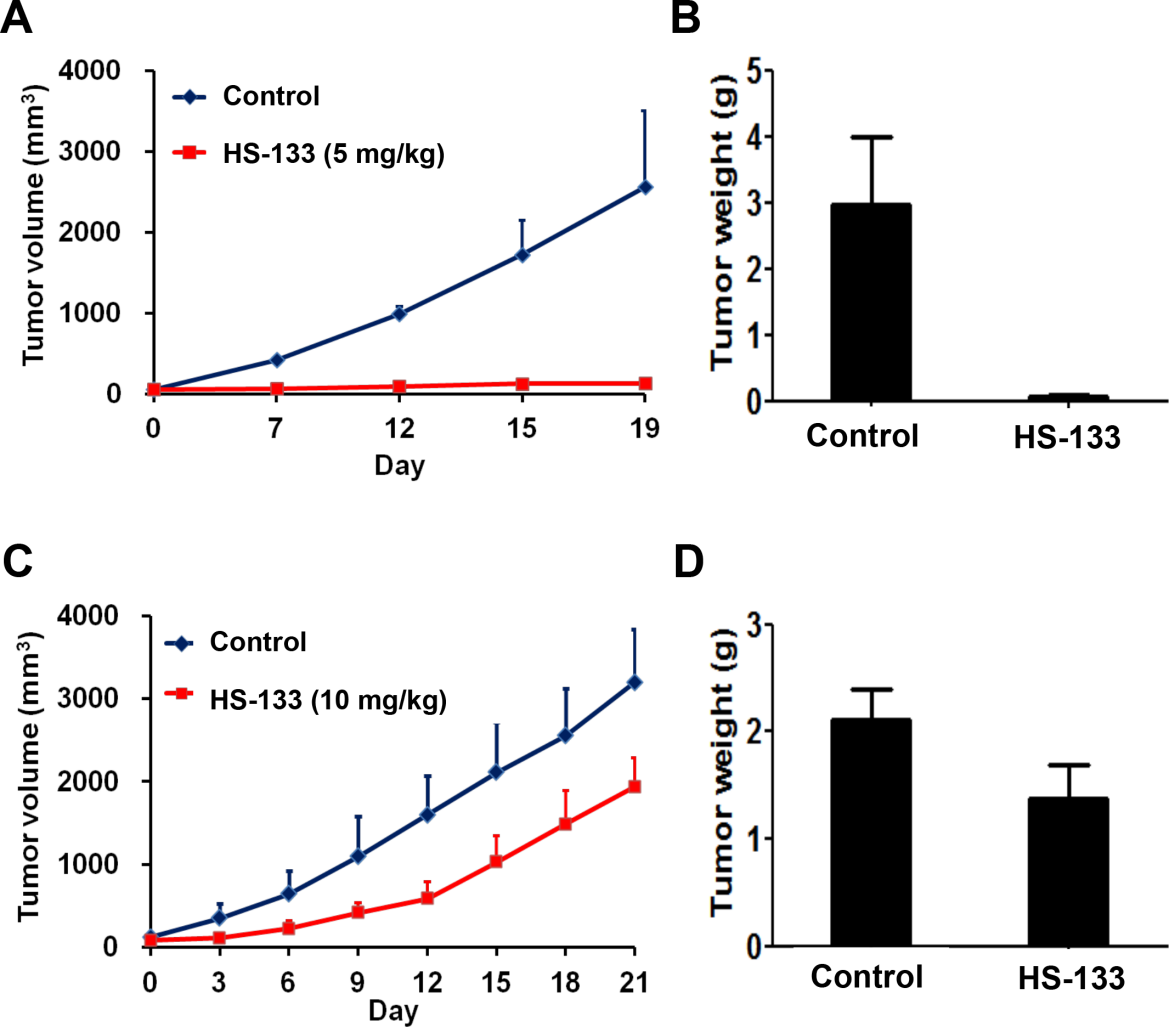
Antitumor activity of HS-133 against SkBr3 xenograft **(A–B)** Mice bearing subcutaneously implanted SkBr3 cells were intratumorally injected with HS-133 (5 mg/kg) twice a week for 19 days. **(C-D)** Mice bearing subcutaneously implanted SkBr3 cells were orally administered with HS-133 (10 mg/kg) daily for 21 days.

We examined the effects of HS-133 using athymic BALB/c nude mice implanted with the SkBr3 cells. HS-133 was injected intratumorally 2 times per week with a dose of 5 mg/kg when tumors reached an average volume of 50–100 mm^3^. As a result, tumor volume and weight were remarkably reduced, showing an antitumor activity in mice treated with HS-133 (Fig. 8A and 8B). When HS-133 was also administered orally with a daily dose of 10 mg/kg for 21 days, it significantly suppressed the tumor growth (Fig. 8C). The average tumor volume of HS-133 treated mice was reduced by about 50% compared to that of control mice (Fig. 8D). To assess the general toxicity, we also measured the body weight change in tumor-bearing mice. The same dose of HS-133 showed no significant change in the body weight (data not shown), suggesting little toxicity of HS-133 at the tested dosage and conditions.

### HS-133 exhibits the good oral bioavailability

**Figure 9:**
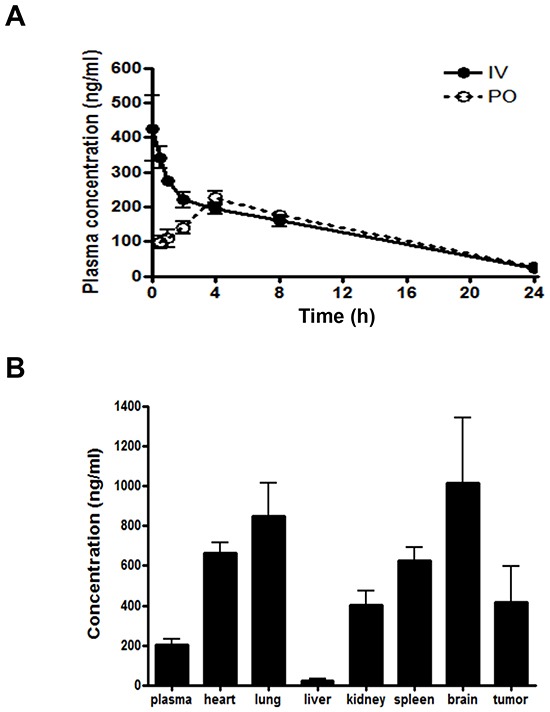
Plasma concentration-time profile and tissue distribution profile of HS-133 following oral (PO) administration or intravenous (IV) administration to mice **(A)** Pharmacokinetic profile of HS-133 after PO administration or IV injection at 5 mg/kg in ICR mice (n=5). The pharmacokinetic parameters of HS-133 are shown in Table 1. **(B)** Variety of tissues concentration of HS-133 following PO administration (10 mg/kg) to SkBr3 xenograft mice.

Extensive preclinical pharmacokinetic evaluation of HS-133 in ICR mice and BALB/c nude mice bearing SkBr3 xenograft has been performed. The plasma concentration-time profile of HS-133 after oral (PO) and intravenous (IV) administration is shown in Fig. 9A. In brief, the peak plasma concentration (C_max_) of HS-133 was 236 ng/mL occurring at approximately 4.8 h post-dose, and the area under the plasma concentration-time curve (AUC) after intravenous and oral administration were 3,410 and 3,260 h×ng/mL, respectively. The bioavailability (F value) of HS-133 was 95.6%; thus, almost all molecules of HS-133 after oral administration were exposed to the systemic circulation system. Clearance (CL) and volume of distribution at the steady-state (V_ss_) after intravenous administration of HS-133 showed to be 1,480 mL/h/kg and 12,000 mL/kg, respectively. The high V_ss_ value of HS-133 may indicate that HS-133 shows a rapid and high distribution to the tissues. Other major pharmacokinetic parameters are shown in Table 1.

**Table 1:** Pharmacokinetics parameters of HS-133 after intravenous and oral administration at a dose of 5 mg/kg in ICR mice (n=5) Data represent mean ± standard deviation (S.D.).

Parameters	*i.v.*	*p.o.*
T_max_ (h)	-	4.8 ± 1.8
C_max_ (ng/mL)	-	236 ± 34
T_1/2, λ_ (h)	6.4 ± 0.92	6.1 ± 1.4
AUC_0–24hr_ (h·ng/mL)	3,190 ± 288	3,000 ± 335
AUC_0-∞_ (h·ng/mL)	3,410 ± 355	3,260 ± 408
CL (mL/h/kg)	1,480 ± 157	-
V_ss_ (mL/kg)	12,000 ± 540	-
MRT (h)	8.2 ± 0.8	9.5 ± 1.9
F (%)		95.6

Various tissue concentrations, such as the heart, lung, liver, kidney, spleen, brain, and tumor in BALB/c nude mice bearing SkBr3 xenograft are shown in Fig. 9B. The ratios of each tissue-to-plasma (K_P_ value) were 3.26, 4.18, 0.11, 1.99, 3.07, 4.99, and 2.06 for the heart, lung, liver, kidney, spleen, brain, and tumor, respectively. Almost all tissues except the liver showed high concentration levels of HS-133 compared to the plasma concentration. Especially, HS-133 in the tumor tissue also showed high concentration level, being over two times the plasma concentration level. Thus, this high concentration level of HS-133 in tumor tissues may be contributed to *in vivo* efficacy in the SkBr3 xenograft model.

## DISCUSSION

The PI3K/Akt pathway is one of the most frequently activated and deregulated pathways in various human cancers [6, 32, 33]. Specifically, over 70% of human breast cancers have a deregulated PI3K/Akt pathway [34]. The high frequency of PI3K/Akt pathway alterations in cancer has led to a surge in the development of PI3K inhibitors. Therapeutic targeting of the PI3K/Akt pathway with small molecule inhibitors may have clinical benefit, either as a single agent in PI3K-addicted cancers or used more broadly in combination with other conventional or targeted therapies. Several classes of inhibitors targeting this pathway, including both the natural and chemically synthesized agents, have been reported and now entered the clinical trials phase. Despite differences in chemical structures, most PI3K inhibitors competitively inhibit ATP binding at the catalytic site [17, 33, 35, 36]. Although the monitoring of intracellular drug delivery and intratumoral distribution can provide information for target therapy, it has remained difficult and proven to still be challenging. Radiolabeling exposes patients to ionizing radiation and results in imaging with poor spatial and temporal resolution. Optical labeling overcomes these disadvantages but suffers from poor tissue penetration, preventing whole-body imaging [37]. Therefore, there is a critical need for noninvasive functional imaging biomarkers that confirm drug delivery and molecular drug activity at the tumor. In an effort to develop potent PI3K inhibitors, which provide an imageable ‘readout’, we screened a large number of novel fluorescent analogs of xanthine [26]. In this study, we describe the detailed pharmacologic characterization of HS-133, which revealed high potency against PI3Kα (0.992 μM) among them. We have shown that HS-133 modulated PI3K/Akt signaling, elicited cell cycle arrest regulating p27, cyclin-dependent kinase (CDK) protein and cyclin D1, and ultimately induced apoptosis through extrinsic and mitochondria-related pathways in breast cancer cells. Our findings are consistent with the results that the PI3K/Akt pathway contributes to the regulation of cell cycle progression, particularly at the G1/S transition [3, 38].

Interestingly, inhibition of the PI3K/Akt pathway may attack tumors by two distinct directions, by blocking the tumor cell growth directly and by inhibiting tumor angiogenesis. It is notable that the PI3K/Akt pathway plays an important role in the production of the key endothelial cell growth factor, VEGF, and in the signaling of the VEGF receptor [33]. HIF-1α plays a central role as the main regulator of the hypoxic transcription response [39], and VEGF is a downstream molecule of HIF-1α [40]. As shown in Fig. 6, HS-133 inhibited not only the expression of HIF-1α and VEGF under hypoxia induced by CoCl_2_ in SkBr3 cells, but also inhibited tube formation of and migration of the endothelial cells. Therefore, we suppose that HS-133 has a potential anti-angiogenic capacity. Although HS-133 is very similar to the pharmacological mechanism of IPD-196, a previously reported PI3K inhibitor, the potency is distinctly different [41]. In addition, the IC_50_ of HS-133 was higher than the other PI3K inhibitor without fluorescence such as HS-104 in breast cancer [42]. Thus, we need to further develop the potent PI3K inhibitors with fluorescent function.

We also demonstrated that orally administered and intratumorally injected HS-133 has therapeutic efficacy against human breast xenografts in mice. To the best of our knowledge, this is the first report of an intratumorally injected PI3K inhibitor with antitumor activity and fluorescent property *in vivo*. Until now, only a few fluorescent inhibitors have been reported. For examples, triapine, which entered phase I and phase II clinical trials as a potent inhibitor of the enzyme ribonucleotide reductase was shown to possess intrinsic fluorescence properties [43]. Yenugonda *et al*. reported that fluorescent CDK inhibitors (VMY-1-101 and VMY-1-103) blocked the proliferation of human breast cancer cells [44]. Histone-deacetylase-targeted fluorescent ruthenium (II) polypyridyl complexes have been shown to display promising anticancer agents for the potential dual imaging and therapeutic application [45]. However, these studies only experimented *in vitro* without *in vivo* experiments. We investigated *in vivo* fluorescence imaging and detected the fluorescence in SkBr3 xenograft models. In addition, surprisingly, we observed that oral administration of HS-133 had a remarkably good bioavailability, and the high concentration level of HS-133 in tumor tissues may be contributed to the *in vivo* efficacy in the SkBr3 xenograft model. In particular, since the level of HS-133 was the highest in the brain tissues, it seems to have the potential for use in a targeted therapy for brain diseases. Furthermore, we observed that HS-133 is more efficient than other previously reported PI3K inhibitors in human breast cancer. For example, although the IC_50_ of HS-104 (4.8 μM) was more effective by approximately 8 folds than HS-133 (32 μM) in SkBr3 cells, tumor growth inhibition was shown at the lower dose of HS-133 compared to HS-104 against SkBr3-bearing xenograft mice (respectively 10 mg/kg/d, 20 mg/kg/d) [42]. The reason why HS-133 showed better effect in our experiments may be due to good oral bioavailability. Recent studies have demonstrated that a number of natural products isolated from the plants (e.g. fruits, vegetables, spices, nuts, legumes, herbs, etc.) also inhibit the PI3K/Akt/mTOR pathway, and exhibit potent anticancer activities. As most of the natural products occur in our every diet day, and are very safe, the results suggest that those natural products may be explored for cancer prevention and treatment [36]. This special issue selects apigenin [46], curcumin [47], cryptotanshinone [48], fisetin [49], indoles (indole-3-carbinol and 3, 3-diindolylmethane) [50], isoflavones (genistein and deguelin) [51], quercetin [52], resveratrol [53], and tocotrienol [54]. Among them, cryptotanshinone is a potential anticancer agent. However, cryptotanshinone has not been in clinical trials for any cancer therapy because of its poor bioavailability. Considering the poor bioavailability of natural products, the good bioavailability of HS-133 can be the best advantage.

In conclusion, we have designed and synthesized HS-133, a fluorescent PI3K inhibitor with potent anti-proliferative, pro-apoptotic capabilities, anti-angiogenic, and anti-tumor effects in human breast cancer. The intrinsic fluorescent properties of HS-133 may be proved to be useful by providing opportunities to study the cellular distribution, delivery and behavior in the biological system of the drugs. These findings lead us to believe that HS-133 may represent to be a promising class of anticancer agents for potential dual imaging and therapeutic applications targeting PI3K/Akt signaling.

## MATERIALS AND METHODS

### Cells and materials

The human breast cancer cell lines SkBr3, T47D, and MCF-7, were purchased from the Korean Cell Line Bank (KCLB, Seoul, Korea). The SkBr3 cells were cultured in Dulbecco’s modified Eagle’s medium (DMEM), and T47D and MCF-7 cells were cultured in Roswell Park Memorial Institute Media 1640 (RPMI-1640), supplemented with 10% fetal bovine serum (FBS) and 1% penicillin/streptomycin. FBS, cell culture media, penicillin-streptomycin, and all other agents used in cell culture studies were purchased from GIBCO (Grand Island, NY). Cultures were maintained at 37°C in a CO2 incubator with a controlled humidified atmosphere composed of 95% air and 5% CO2. Human umbilical vein endothelial cells (HUVECs) were grown in a gelatin coated 75 cm2 flask in a M199 medium containing 20 ng/ml basic fibroblast growth factor (bFGF), 100 U/ml heparin and 20% FBS at 37°C. Propidium iodide (PI), 3-(4, 5-dimethylthiazol-2-yl)-2, 5-diphenyltetrazolium bromide (MTT), and proteinase K were purchased from Sigma-Aldrich (St. Louis, MO). RNase A was purchased from Qiagen (Valencia, CA).

#### 7-(3-Fluorophenyl)-1, 3-dimethyl-1*H*-purine-2, 6(3*H*,7*H*)-dione

To a solution of theophylline (4.01 g, 22.2 mmol), 3-fluorophenylboronic acid (4.04 g, 28.9 mmol), and copper(II) acetate (4.84 g, 26.6mmol) in dichloromethane (40 mL) was added pyridine (4.5 mL, 55.5 mmol), and the entire solution was left stirring at 40°C for 5 h. The solution was then filtered through a pad of celite, and the filtrate was concentrated under a reduced pressure. The filtrate was washed with dichloromethane, H_2_O and dried (MgSO_4_). The solvent was concentrated *in vacuo* and 7-(3-Fluorophenyl)-1, 3-dimethyl-1*H*-purine-2, 6(3*H,*7*H*)-dione (44%) was purified by recrystallization with MeOH; ^1^HNMR δ(300 MHz, CDCl_3_); 3.51 (3H, s), 3.62 (3H, s), 7.14 (1H, t), 7.20 (1H, t), 7.24 (1H, t), 7.44 (1H, q), 7.82 (1H, s); ^13^C NMR δ (75 MHz, CDCl_3_): 28.17, 29.90, 106.99, 112.67, 115.99, 120.66, 135.82, 141.07, 149.70, 151.41, 154.26, 160.82, 164.11; HRMS (EI+) m/z calcd for C_13_H_12_FN_4_O_2_[M+H]^+^: 275.0944, found: 275.0945

#### *N*-(4-(7-(3-Fluorophenyl)-1, 3-dimethyl-2, 6-dioxo-2, 3, 6, 7-tetrahydro-1*H*-purin-8-yl) phenyl)acetamide

To a solution of 1, 3-dimethyl-7-*N*-(3-fluorophenyl)xanthine (1.19 g, 4.3 mmol), *N*-(4-bromophenyl) acetamide (1.39 g, 6.5 mmol), copper (I) iodide (2.48 g, 13.0 mmol) and palladium (II) acetate (291 mg, 1.3 mmol) in DMF was added cesium carbonate (3.54 g, 10.9 mmol) and the solution was set in a sealed tube. Reactions were carried out at about 140°C for 24 h under nitrogen purged status. The solution was filtered through celite to remove palladium. The filtrate was washed with dichloromethane, H_2_O and dried (MgSO_4_). The solvent was concentrated *in vacuo* and the residue was purified by flash column chromatography (CH_2_Cl_2_:MeOH, 20:1) to produce the desired *N*-(4-(7-(3-Fluorophenyl)-1, 3-dimethyl-2, 6-dioxo-2, 3, 6, 7-tetrahydro-1*H*-purin-8-yl)phenyl)acetamide (48%); ^1^HNMR δ(300 MHz, CDCl_3_): 2.14 (3H, s), 3.43 (3H, s), 3.67 (3H, s), 7.02 (1H, m), 7.10 (1H, d), 7.17 (1H, m), 7.37(5H, dd); ^13^C NMR δ (75 MHz, CDCl_3_): 24.64, 28.00, 29.88, 108.97, 115.67, 116.63, 119.06, 123.40, 129.98, 130.51,,136.85, 139.85, 148.71, 150.80, 151.55, 154.30, 160.76, 164.07, 168.28; HRMS (EI+) m/z calcd for C_21_H_19_FN_5_O_3_ [M+H] ^+^: 408.1472, found: 408.1465.

### Cell viability assay

Cell viability was performed by an MTT assay. Briefly, the SkBr3, T47D, and MCF-7 cells were plated at a density of 1-5 × 10^3^ cells/well in a 96-well plate for 48 h. The medium was removed, and the cells were treated with either DMSO as control or various concentrations of HS-133. The final concentration of DMSO in the medium was ≤ 0.1% (v/v). After the cells were incubated for 48 h, 20 μl of MTT solutions (2 mg/ml) was added to each well for another 4 h at 37°C. The formazan crystals that formed were dissolved in DMSO (200 μl/well) via constant shaking for 5 min. The plate was then read on a microplate reader at 540 nm. Three replicate wells were used for each analysis. The median inhibitory concentration (IC_50_, defined as the drug concentration at which cell growth was inhibited by 50%) was assessed from the dose-response curves.

### Fluorescent detection of HSS-133 by confocal microscopy

The SkBr3 cells were plated on18-mm cover glasses in DMEM medium and incubated for 24 h so that approximately 70% confluence was reached. The cells were then incubated in the presence or absence of 10 μM HS-133 for 4 h. After washing with phosphate buffered saline (PBS) three times, each slide was covered with DABCO (Sigma-Aldrich) and observed using a confocal laser scanning microscope (Olympus, Tokyo, Japan).

### Western blotting

The cells were washed three times with ice-cold PBS before lysis, and were lysed with a buffer containing 1% Triton X-100, 1% Nonidet P-40 (NP-40), as well as protease and phosphatase inhibitor cocktails (GenDEPOT, Barker, TX). Equal amounts of protein were separated by 10% sodium dodecyl sulfate-polyacrylamide gel electrophoresis (SDS-PAGE) and transferred onto the polyvinylidene fluoride (PVDF) membranes (Millipore, Bedford, MA). Immunostaining was performed by incubating the blots with primary antibodies, followed by horseradish peroxidase (HRP)-conjugated secondary antibody and detected with an enhanced chemiluminescence (ECL) plus system (Amersham Biosciences, Piscataway, NJ). The primary mouse antibodies against the following factors were used: p27 (Cell Signaling Technologies, Danvers, MA), cyclin D1, hypoxia-inducible factor-1α (HIF-1α, BD Biosciences, SanJose, CA), VEGF (Novus Biologicals, Littleton, CO) and β-actin (Abcam, Cambridge, MA). The primary rabbit polyclonal antibodies against the following proteins were also used: cleaved caspase-3, poly (ADP-ribose) polymerase protein (PARP), p-Akt, Akt, p-mTOR, mTOR, p-p70S6K, p70S6K, p-4E-BP1, 4E-BP1 (Cell Signaling Technologies), cleaved caspase-8, procasepase-9 (Santa Cruz Biotechnology, Santa Cruz, CA), and α-tubulin (Abcam). The secondary antibodies were purchased from Amersham Biosciences.

### Cell cycle analysis

The SkBr3 cells were plated in 100 mm-diameter culture dishes. On the following day, the cells were treated with various concentrations of HS-133 or 0.1% DMSO for 24 h. The floating and adherent cells were collected and fixed in cold 70% ethanol at 4°C overnight. After washing, the cells were subsequently stained with 50 μg/ml propidium iodide (PI) and 100 μg/ml RNase A for 1 h in the dark and subjected to a flow cytometric analysis to determine the percentage of cells at specific phases of the cell cycle. A flow cytometric analysis was performed using a FACSCalibur flow cytometer (Becton Dickinson, San Jose, CA) equipped with a 488 nm argon laser. The events were evaluated for each sample and the cell cycle distribution was analyzed using Cell Quest software (Becton Dickinson). The results were presented as the number of cells versus the amount of DNA, as indicated by the intensity of a fluorescence signal. All the experiments were conducted three times.

### Terminal deoxynucleotidyl transferase–mediated nick end labeling (TUNEL) assay

The SkBr3 cells were plated on 18-mm cover glasses in DMEM medium and incubated for 24 h so that approximately 70% confluence was reached. The cells were then incubated in the presence or absence of 50 μM HS-133, washed twice with PBS, and fixed in an acetone: methanol solution (1:1) for 10 min at −20°C. The stained cells were examined for a fluorescence of nuclear fragmentation. Terminal deoxynucleotidyl transferase–mediated nick end labeling (TUNEL) was performed using the TUNEL kit (Millipore, Billerica, MA).

### Tube formation assay

A 10 mg/ml (200 μl) of Matrigel (BD Biosciences) was polymerized for 30 min at 37°C. HUVECs were suspended in M199 (2% FBS) medium containing VEGF (50 ng/ml) at a density of 2.5 × 10^5^ cells/ml, and 0.2 ml of cell suspension was added to each well coated with Matrigel, together with or without the indicated concentrations of HS-133 for 20 h. The morphological changes of the cells and tubes formed were observed under a phase-contrast microscope and photographed at 200× and 400× magnification.

### Wound migration assay

HUVECs plated on 60 mm-diameter culture dishes at 90% confluence, were wounded 2 mm in width with a sterile razor blade and marked at the injury line. After wounding, the peeled off cells were removed with a serum-free medium and further incubated in M199 with 2% FBS, 1 mM thymidine (Sigma-Aldrich), HS-133 (50 nM) and/or VEGF (50 ng/ml). HUVECs were allowed to migrate for 18 h and were rinsed with a serum-free medium, followed by fixing with absolute methanol and staining with Giemsa (Sigma-Aldrich). Migration was quantitated with counting the number of cells that moved beyond the reference line.

### Enzyme-linked immunosorbent assay (ELISA)

The amount of VEGF secreted into the media was measured by sandwich ELISA. ELISA plates (Nunc, Roskilde, Denmark) were coated with 100 μl of 2 μg/ml anti-VEGF (R&D Systems, Minneapolis, MN) antibody in PBS for 24 h at 25°C. The plates were washed with PBS containing 0.1% Tween-20 and incubated for 1 h at 25°C with 200 μl/well of 1% bovine serum albumin (BSA, Sigma-Aldrich) in PBS. The conditioned medium or various concentrations of recombinant human VEGF were incubated for 2 h at 25°C with 100 μl of 75 ng/ml biotinylatedanti-VEGF antibody, and the plates were washed and further incubated for 30 min with 100 μl of HRP-conjugated streptavidin (Vector Laboratories). After washing, the reaction was stopped by adding 50 μl of 2 N H_2_SO_4_. The absorbance at 450 nm was measured with a 96-well plate reader.

### Tumor xenograft studies

To establish SkBr3 tumor xenograft in mice, the SkBr3 cell line was grown in a culture, detached by trypsinization, washed and resuspended in PBS. Six-week old athymic BALB/c nude mice (Central Lab. Animal Inc. Seoul, Korea) were injected with 5 × 10^6^ cells in the right flank of each mouse to initiate tumor growth. After reaching a tumor volume of 50–100 mm^3^, mice were randomly divided into two groups, each having six mice. Mice in the control group were fed with a 0.2 mL vehicle (DMSO:PEG400:saline = 5:50:45) by oral gavage daily and the second group with 10 mg/kg dose of HS-133 in 0.2 mL daily for 21 days. For intratumoral administration, HS-133 was injected intratumorally with 2 times per week with a dose of 5 mg/kg when tumors reached an average volume of 50–100 mm^3^. The body weight and tumor size were recorded twice per week. The tumor size was calculated by 0.5 × long axis × (short axis) ^2^. Animal care and experimental procedures were conducted in accordance with the Guide for Animal Experiments by the Korean Academy of Medical Sciences, and the protocols used to this study were approved by the Institutional Animal Care and Use Committee in Inha University Hospital (Approval No. 110802-105).

### *In vivo* imaging study

Six-week old athymic BALB/c nude mice (Central Lab. Animal Inc. Seoul, Korea) were injected with 5 × 10^6^ SkBr3 cells in the right flank of each mouse to initiate tumor growth. HS-133 was injected via an intratumoral injection in the SkBr3 tumor bearing mice. The mice were anesthetized with intraperitoneal (IP) administration of ketamin/rumpun, then, spectral fluorescence images were obtained with the Maestro™ In-Vivo Fluorescence Imaging System (CRi, Inc., Woburn, MA). The spectral fluorescence images consisting of HS-133 (5 mg/kg) and autofluorescence spectra were then unmixed based on their spectral patterns using commercial software (Maestro software, CRi).

### Pharmacokinetic study in mouse

Eight-week old ICR mice (23–25 g) were orally given a 5 mg/kg dose of HS-133 in DMSO:PEG400:saline = 5:50:45. Blood samples (approximately 0.15 mL) were collected from each mouse (n = 3 mice per time point) by a retro-orbital bleed or terminal cardiac puncture under isoflurane anesthesia. Blood samples were collected in tubes containing K_2_-EDTA as the anticoagulant, pre-dose and at 0.5, 1, 2, 4, 8, and 24 h post-dose. Samples were centrifuged, and the plasma was collected and stored at −80°C until analysis. Total concentrations of HS-133 were determined by LC-MS/MS, following plasma protein precipitation with acetonitrile, and injection of the supernatant onto the column, a Hypersil Gold C18 column (50 mm × 2.1 mm, 3μmparticle size; Thermo, Waltham, MA). The Agilent 1260 HPLC and Agilent 6460 triple quadrupole mass spectrometry system (Agilent Inc., Santa Clara, CA) were used for the LC-MS/MS assay. The mobile phase consisted of acetonitrile and 10 mM ammonium formate buffer (80:20, v/v), at a flow rate of 0.3 mL/min. The lower and upper limits of quantitation of the assay were 0.005 and 10 μg/mL, respectively. The total run time was 1.5 min and the ionization was conducted in the positive ion mode.

The heart, lung, liver, kidney, brain, spleen, and tumor were collected at 30 min post-dose from 3 different animals at each time point, rinsed with ice-cold saline, weighed and stored at −80°C until analysis. For HS-133 quantification, mouse tissues were homogenized with three times the volume of PBS. The homogenates were extracted by protein precipitation with acetonitrile. LC-MS/MS analysis was conducted as described for the plasma. The homogenate concentrations were converted to tissue concentrations for the calculations of each tissue-to-plasma ratio.

### Statistical analysis

Data are expressed as the mean ± S.D. Statistical analysis was performed using ANOVA and an unpaired Student′s t-test. A p-value of < 0.05 was considered statistically significant. Statistical calculations were performed using SPSS for Windows Version 10.0 (SPSS, Chicago, IL).
